# Alkali-Fused Coal Gasification Fine Slag for Simulated Phenolic Wastewater Treatment: Adsorption–Oxidation Performance and Mechanistic Investigation

**DOI:** 10.1021/acsomega.6c05846

**Published:** 2026-08-13

**Authors:** Wenxian Hu, Peijun Liu, Suqian Gu, Ruijun Yan, Jianfei Li, Shengli An, Yifan Chai

**Affiliations:** † School of Metallurgical Future Technology, 177540 Inner Mongolia University of Science and Technology, Baotou 014010, China; ‡ Key Laboratory of High Efficiency Green Metallurgy of Bayan Obo Rare Earth Iron and Niobium Associated Ore in Inner Mongolia Autonomous Region, Baotou 014010, China; § Inner Mongolia Autonomous Region Carbon Neutral Collaborative Innovation Center, Baotou 014010, China

## Abstract

Coal gasification fine slag (CGFS) is a large amount of industrial solid waste produced by the coal chemical process. Phenol in coking wastewater is a kind of refractory and highly toxic organic pollutants. In order to solve the two types of environmental problems simultaneously, porous active materials (CGFS-AFAC-800) were prepared by the NaOH alkali fusion method at 800 °C using CGFS as the raw material and applied to the efficient removal of phenol in simulated coking wastewater. The key adsorption parameters were optimized by the single factor experiment and orthogonal experiment. The adsorption performance and mechanism were studied by adsorption kinetics, isothermal adsorption, and adsorption thermodynamics. The results showed that the optimum adsorption conditions were as follows: activated material dosage 10 g·L^–1^, adsorption time 60 min, temperature 40 °C, initial pH = 3, H_2_O_2_ dosage 5 mM, and the removal rate of phenol was up to 99.90%. The adsorption kinetics conformed to the pseudo-first-order kinetic PFO model, *R* ^2^ = 0.9937, *K*_1_ = 0.07 min^–1^, *q*_e_ = 13.30 mg·g^–1^. The isothermal adsorption process conformed to the Freundlich model, *R* ^2^ = 0.9950, *n* >1 at 35 °C. The thermodynamic analysis results show that the adsorption reaction is a spontaneous endothermic physical adsorption process. This study realized the high-value conversion of coal gasification fine slag to high-performance activated material, which not only alleviated the pressure of industrial solid waste disposal but also provided a low-cost technical solution for phenol-containing wastewater treatment, with significant environmental and economic benefits.

## Graphical abstract

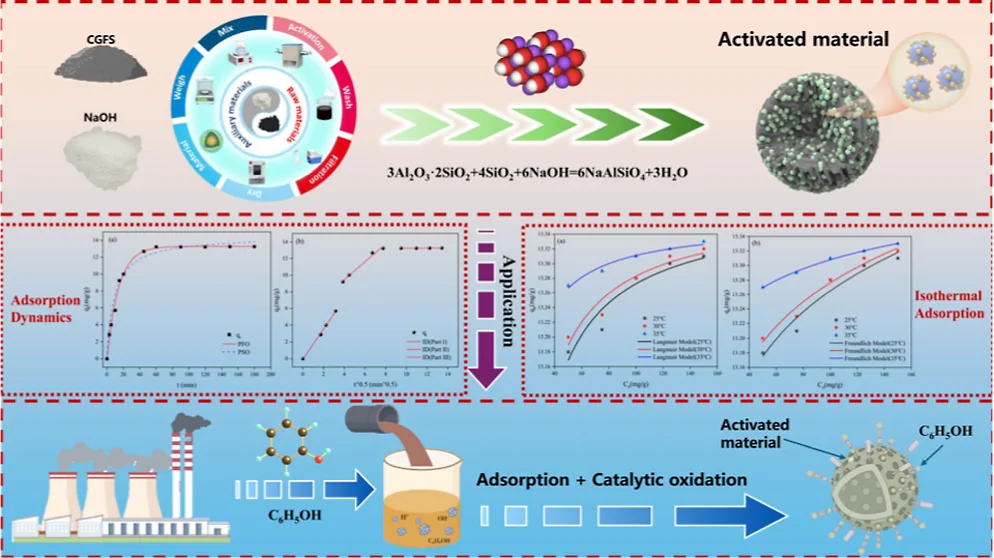

## 1 Introduction

China’ s iron and steel industry is an important pillar industry of the national economy. The coking wastewater generated in the coking production process is huge,^1,2^ which is a typical type of highly polluting industrial wastewater. Statistics indicate that producing one ton of coke generates 0.4 to 0.9 m^3^ of wastewater, and the yearly coking wastewater production nationwide amounts to roughly 300 million tons.^3^ Phenol^4^ is a typical refractory organic pollutant produced in the production process of coal chemical, coking, pharmaceutical, and other industries. It has strong toxicity, carcinogenicity, and bioaccumulation. At present, the treatment methods of phenol wastewater mainly include biodegradation, chemical oxidation, membrane separation, and adsorption. Among them, the biodegradation method^5^ has poor tolerance to high-concentration phenol and long treatment cycle; the chemical oxidation method^6^ is easy to produce secondary pollution and has a high operating cost; the membrane separation method^7^ has problems such as membrane fouling and large investment scale.

As the core means of clean and efficient utilization of coal, coal gasification has gained extensive adoption throughout the coal chemical industry, but this process will produce a large amount of solid waste^8^—coal gasification fine slag (CGFS).^9^ According to statistics, China’s annual coal gasification fine slag emissions exceed 10 million tons.^10^ If this kind of waste is only simply stored or landfilled, on the one hand, it will cause a large amount of land resources to be occupied, and on the other hand, there are secondary pollution risks such as heavy metal leaching and dust diffusion, which has become an important obstacle to the green and sustainable development of the coal chemical industry. It is worth noting that the chemical composition of coal gasification fine slag has the natural advantage of being converted into adsorption materials: the total mass fraction of SiO_2_ and Al_2_O_3_ in its inorganic components is as high as 72.75%, which can form a porous structure through activation and reconstruction. The fixed carbon content is 80.49%, which can provide basic adsorption sites for the adsorption process. The conversion of this industrial solid waste into activated material for phenol wastewater treatment^11^ can not only solve the problem of solid waste disposal but also reduce the cost of wastewater treatment.

However, due to the lack of surface functional groups and other factors, the original coal gasification fine slag has low adsorption capacity and poor removal efficiency for phenol and cannot be directly used as an efficient activated material. In order to improve its adsorption performance, researchers have developed a variety of activation technologies such as high temperature calcination, acid leaching activation, and microwave activation.^10^ However, these methods generally have defects such as high energy consumption (such as high temperature calcination requires more than 1000 °C), easy to produce secondary pollution (such as acid leaching to produce acidic wastewater), and complex processes, which limit their large-scale application. As a mild and low-cost solid waste activation technology, Hu et al.^12^ reconstructed the mineral phase through the reaction of alkali with SiO_2_ and Al_2_O_3_ in coal gasification fine slag to form porous structures such as nepheline and activated the residual carbon to significantly improve the adsorption activity of the material, providing a theoretical basis for the preparation of high-efficiency activated material from coal gasification fine slag. The improvement of the adsorption performance of coal gasification fine slag by the alkali fusion method is mainly due to the double-effect synergy of mineral phase reconstruction and residual carbon phase etching. Under high-temperature conditions, NaOH, as a strong alkali activator, can not only destroy the lattice structure of dense minerals such as quartz and kaolinite in raw materials but also generate nepheline porous crystal phase to build a mineral-based pore network and can oxidize and etch residual carbon components to achieve carbon phase porosity. Both of them contribute to the formation of microporous–mesoporous hierarchical pore structure, which significantly improves the degree of pore development and the number of adsorption sites. At the same time, they can regulate the surface oxygen-containing functional groups and charge distribution, supplemented by π–π stacking and weak electrostatic interaction to enhance the adsorption of phenol. The overall adsorption process is dominated by physical action.

The activation material CGFS-AFAC-800 used in this study was prepared by the NaOH alkali fusion activation method. The preparation process was carried out strictly according to the optimal parameters after the previous optimization:^12^ CGFS was used as a raw material, mixed according to the activation ratio of 0.4, dried and placed in a horizontal tube furnace, and heated to 800 °C for 30 min at 10 °C/min in a N_2_ protective atmosphere. The product was washed to neutral, dried at 105 °C, and ground to obtain the target activation material CGFS-AFAC-800. The research content focuses on the optimization of material preparation methods, XRD/SEM/BET/FTIR and other physical and chemical characterization, phase reconstruction, and pore structure evolution mechanism, which fully supports the material properties and preparation repeatability of CGFS-AFAC-800.^12^ In this manuscript, based on the material obtained under the optimal preparation conditions, the extensibility application and mechanism research based on the above preparation process are carried out. The core goal is to solve the process problem of efficient treatment of phenol wastewater. As a single factor test and orthogonal test system, the key parameters such as activated material dosage, adsorption time, temperature, solution pH, and H_2_O_2_ dosage were optimized. Combined with the pseudo-first-order/pseudo-second-order kinetic model, Langmuir/Freundlich isotherm model, and thermodynamic analysis, the adsorption mechanism of phenol on the material was revealed. The preparation parameters of materials have not been subjected to secondary optimization. The research results can provide a theoretical basis for the resource utilization of coal gasification fine slag.

## 2 Experiment Section

### 2.1 Raw Material and Reagents

The raw material used in this study is coal gasification fine slag (CGFS) from a coal chemical enterprise in the Inner Mongolia. The raw materials were dehydrated and dried for use. The basic physical and chemical properties such as chemical composition, industrial analysis, and elemental composition were detailed in the previously published work.^12^ The basic chemical reagents such as NaOH and phenol used in the experiment are analytically pure reagents.^13^

### 2.2 Phenol Removal in Simulated Phenolic Wastewater

(1) Single Factor Design

Five beakers of A, B, C, D, and E were prepared, and five beakers were placed in each group. A total of 100 mL of simulated coking wastewater with a phenol concentration of 100 mg/L was added to each beaker. By changing the dosage of CGFS-AFAC-800 activated material, adsorption time, temperature, initial pH value of the solution, and one of the variables of H_2_O_2_ dosage, fixing other adsorption conditions unchanged, five groups of single-factor experiments were carried out. The supernatant was taken after stirring at 130 rpm in a digital water bath, the absorbance was measured, and the adsorption capacity and removal rate of phenol were calculated. Each experiment was repeated three times, and the average value was taken.

The concentration of phenol solution after filtration was detected by an ultraviolet spectrophotometer,^14^ and the calibration curve of absorbance-volatile phenol content is shown in Figure 1.^12^ After degradation, the phenol concentration, adsorption capacity (q_t_), and removal rate (η) were calculated according to Formula [Disp-formula eq1], Formula [Disp-formula eq2],^15^ and Formula [Disp-formula eq3],^16^ respectively.

**Figure 1:**
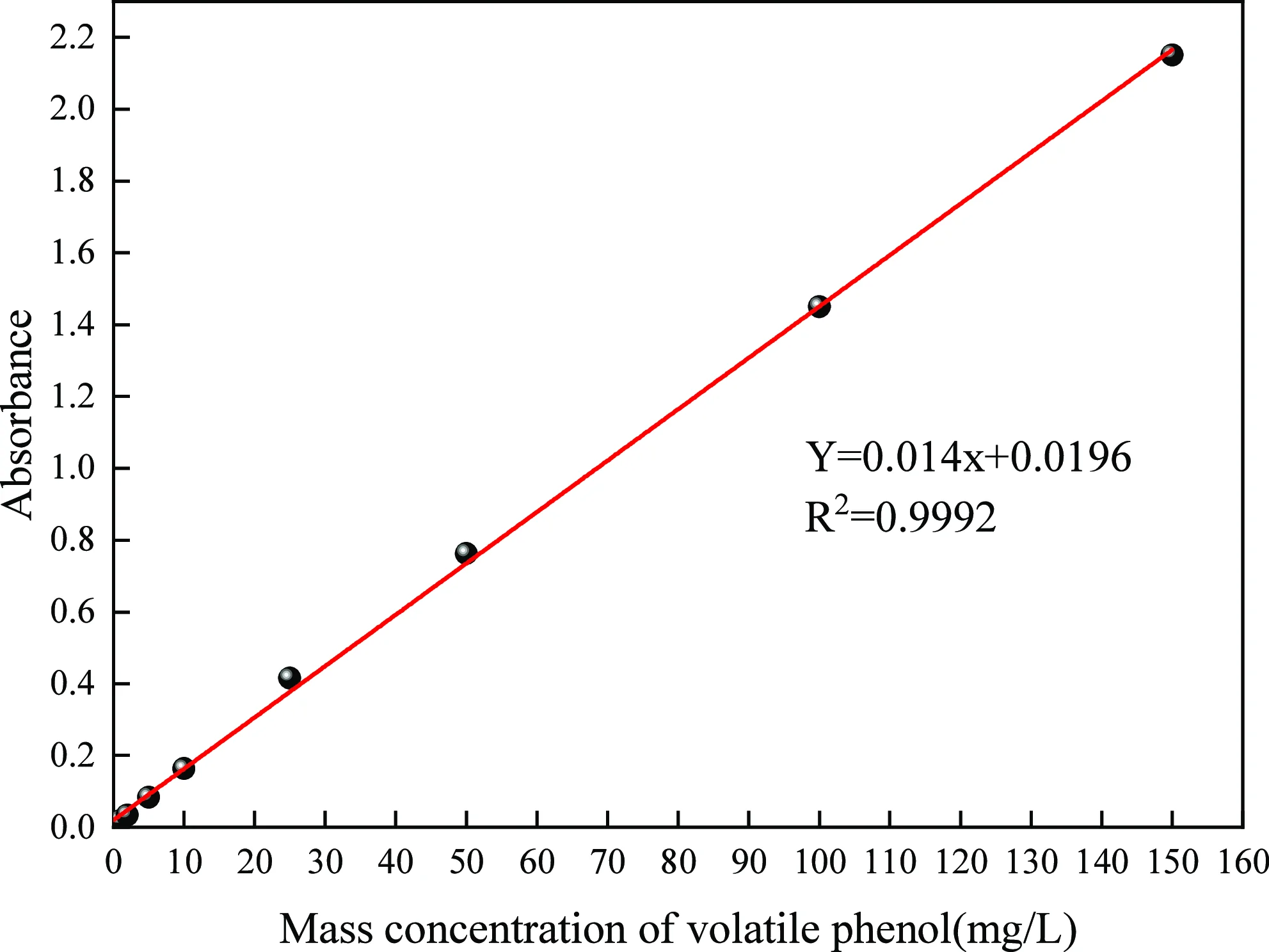
Calibration curve of sample absorbance versus volatile phenol content.

The basic data statistical calculations and thermodynamic parameter calculations of this study were completed by Microsoft Excel software. The nonlinear fitting of the adsorption kinetic model and isothermal adsorption model, the drawing of relevant charts in the full text experiment, and the range analysis of the orthogonal experiment were all completed by Origin 2021 software. In the process of model fitting, the nonlinear least-squares method is used to optimize the parameters, and the determination coefficient *R* ^2^ is used as the core evaluation on the basis of the fitting effect.

Reprinted from ref 12, Figure 1, Copyright (2025), with permission from Elsevier.

1 $$\rho =\frac{A-0.0196}{0.014}$$

where ρ is the mass concentration of volatile phenols in the sample, mg·L^–1^; *A* is the absorbance displayed on the spectrophotometer; 0.014 is the absorbance coefficient, L·(mg·cm)^−1^.

2 $$q_{t}=\frac{C_{0}-C_{t}}{m}V$$

3 $$\eta =\frac{C_{0}-C_{t}}{C_{0}}\times 100\%$$

where *C*_0_ (mg·L^–1^) is the initial concentration of the simulated phenol wastewater solution; *C*_t_ (mg·L^–1^) is the concentration of the simulated phenol wastewater solution at time *t*; *V* (L) is the volume of the simulated phenol wastewater solution; and *m* (g) is the dosage of the activated material.

(2) Orthogonal Design

The orthogonal test table is given in Table 1.

**Table 1:** Orthogonal Test Table

factor level	addition (g)/A	time (min)/B	temperature (°C)/C	pH/D	H_2_O_2_ (mM)/E
1	0.25	30	30	1	0
2	0.5	60	40	3	5
3	0.75	90	55	5	10
4	1.0	120	70	7	15

### 2.3 Adsorption Kinetics Experiments

Adsorption kinetics experiments were used to explore the effects of adsorption time *t* and initial concentration of solution *C*_0_ on adsorption capacity. Specifically, according to the change of adsorption capacity *q*_*t*_ with time *t*, the driving factors affecting the adsorption rate and adsorption equilibrium time can be judged. In the adsorption kinetics experiment, the CGFS-AFAC-800 activated material prepared by the experiment was used as the activated material, and it was mixed with the target concentration of phenol solution according to the solid–liquid ratio of 1.0 g/100 mL. The beaker containing the reactants was placed in a water bath and stirred repeatedly at 130 rpm at 40 °C. The solution samples were taken at different time points between 0 and 180 min, and the absorbance A of the solution was measured according to the above method. After the absorbance A is measured, it can be substituted into Formulas [Disp-formula eq1], 2, and 3 to calculate the corresponding phenol adsorption amount *q*_*t*_ and removal rate η at time *t*. Finally, the relationship between *q*_*t*_ and *t* is drawn.

For the selection of adsorption kinetic models, most studies use the classical pseudo-First-Order kinetic model (PFO) and pseudo-Second-Order kinetic model (PSO) to simulate the kinetic data. These two models are widely used in various solid–liquid adsorption systems, such as porous solid media with biomass and nanomaterials as the activated material and adsorption systems with heavy metals, drugs, or pollutants as adsorbates.^17^ The expressions of PFO and PSO models are divided into linear and nonlinear methods, both of which can obtain adsorption kinetic parameters. However, transforming the dynamic data into the linear form of the model will change the error structure of the fitting results and bring more uncertainty and deviation, while using the nonlinear equation will avoid the above problems. Therefore, in this study, the nonlinear form of the above kinetic model was selected when fitting the adsorption kinetics.^17^ The pseudo-first-order kinetic model considers that the adsorption process of the activated material to the pollutant is based on the membrane diffusion theory.^18^ The reaction between the adsorbate molecule and the adsorption site on the solid surface is independent, and the reaction rate is controlled by the diffusion step. The number of active sites on the activated material surface or the concentration of the solution is the only influencing factor,^19^ and the nonlinear expression is shown in Formula [Disp-formula eq4]. The core theoretical assumption of the quasi-second-order kinetic model is that the rate of adsorption reaction is dominated by the chemical process. During the adsorption process, the electron transfer or sharing occurs between the adsorbent and the adsorbate molecules, and the formation of chemical bonds is the main driving force for the adsorption. The overall adsorption rate depends on both the total amount of active sites on the adsorbent surface and the pollutant concentration level in the solution.^20^ The nonlinear expression is shown in Formula [Disp-formula eq5].

4 $$q_{t}=q_{e}\left(1-e^{-k_{1}t}\right)$$

5 $$q_{t}=\frac{{q_{e}}^{2}k_{2}t}{1+q_{e}k_{2}t}$$

where *t* (min) is the reaction time; *q*_e_ (mg/g) is the adsorption amount at equilibrium; *q*_*t*_ (mg/g) is the adsorption amount of the sample at time *t*; *k*_1_(1/min) and *k*_2_(g/ (mg·min)) are the adsorption rate constants of the pseudo-first-order and second-order kinetic models, respectively, and their values are related to temperature.

The above PFO and PSO models describe the influencing factors of the adsorption reaction rate, but they cannot reflect the specific diffusion of the adsorbate on the activated material. In the ID model, the adsorption process is divided into three stages: membrane diffusion, intraparticle diffusion, and adsorption equilibrium. Therefore, the intraparticle-diffusion model (ID) is introduced to further explain the migration and transformation process of pollutants inside the activated material. Among them, the slower diffusion link is the limiting step of the adsorption rate,^21^ and the mathematical expression of the model is Formula [Disp-formula eq6].

6 $$q_{t}=k_{id}t^{1/2}+c$$

where *k*_id_(mg/(g·min^1/2^)) is the intraparticle diffusion constant; *c*(mg/g) represents the influence of the boundary layer on the adsorption rate.^22^

### 2.4 Isothermal Adsorption Experiment

In this study, multiple sets of temperature gradients were set up to carry out isothermal adsorption experiments. The equilibrium concentration *C*_*e*_ of the solution at each temperature was measured, and the equilibrium adsorption capacity *q*_e_ corresponding to the adsorbent was obtained. Phenol solutions with different initial concentrations were prepared, and the volume of phenol solution used in each adsorption reaction was 100 mL. The activated material and phenol solution were mixed in a beaker according to a certain solid–liquid ratio, and the adsorption reaction was carried out in a water bath. The temperature of the water bath was set at 25, 30, and 35 °C, respectively, and the rotation speed was 130 rpm. The adsorption time was 60 min to reach equilibrium. After the reaction, the supernatant of phenol solution was taken to measure the absorbance, and the equilibrium concentration *C*_e_, equilibrium adsorption capacity *q*_e_ and phenol removal rate η were calculated according to formulas [Disp-formula eq1], 2, and 3. With *C*_e_ as the abscissa and *q*_e_ as the ordinate, the relationship between the two is plotted. Generally, in the solid–liquid equilibrium adsorption reaction system, the adsorption isotherm can reflect the surface properties, pore structure, and liquid–solid intermolecular force of the solid activated material. In this study, Langmuir and Freundlich adsorption isotherm models were used to describe the adsorption behavior of phenol on the CGFS-AFAC-800 activated material.

(1) Langmuir Isothermal Adsorption Model

The Langmuir model assumes that the molecular layer of the activated material is not more than one layer, and the adsorption occurs when the adsorbate molecule collides with the adsorption site on the surface of the activated material, and each molecule only occupies one adsorption site. The adsorption energy of each adsorption site is the same, that is, the adsorption heat is equal, and there is no interaction between the adsorbate molecules.^23^ The isothermal adsorption model is suitable for chemical adsorption and localized physical adsorption on a monolayer, energy-uniform surface. The nonlinear expression is shown in formula [Disp-formula eq7].

7 $$q_{e}=\frac{q_{m}K_{L}C_{e}}{1+K_{L}C_{e}}$$

8 $$R_{L}=\frac{1}{1+K_{L}C_{0}}$$

where *q*_m_ (mg/g) is the maximum adsorption capacity of Langmuir; *q*_e_ (mg/g) is the equilibrium adsorption capacity; *k*_*L*_ (L/mg) is the Langmuir equilibrium constant. *R*_L_ is the adsorption isotherm constant.^24^

(2) Freundlich Isothermal Adsorption Model

The Freundlich isothermal adsorption model assumes that the adsorption sites on the surface of the solid-activated material are not uniform, the adsorbate is enriched in some areas of the surface of the activated material to form an adsorption layer, and the distribution of adsorption energy is not equal. The adsorption process of the heterogeneous surface and multimolecular layer in a nonideal adsorption reaction is described,^25^ and its expression is shown in formula [Disp-formula eq9]:

9 $$q_{e}=K_{F}{C_{e}}^{1/n}$$

where *K*_*F*_ (mg/g­(L/mg)^1/*n*^) is the Freundlich constant, and *n* is the Freundlich nonuniformity factor.^26^

### 2.5 Adsorption Thermodynamics

By calculating the adsorption free energy (Δ*G°*), enthalpy (Δ*H*°), and entropy (Δ*S*°), the degree of adsorption reaction and the driving force of the reaction can be analyzed. The calculation of the above three parameters is mainly based on the ideal gas state equation, van’ t Hoff equation, and Gibbs–Helmholtz equation,^27^ formulas [Disp-formula eq10], 11, and 12.

10 $$\Delta G^{{}^{\circ}}=-RT\;\ln K{}^{\circ}$$

11 $$\Delta G^{{}^{\circ}}=\Delta H^{{}^{\circ}}-T\Delta S^{{}^{\circ}}$$

12 $$\ln K{}^{\circ}=-\frac{\Delta H^{{}^{\circ}}}{RT}+\frac{\Delta S^{{}^{\circ}}}{R}$$

where *T* (*K*) is the absolute temperature of thermodynamics; *R* (8.314 J·mo1^–1^·K^–1^) is a general gas constant; and *K*° (L/g) represents the adsorption thermodynamic equilibrium constant.

## 3 Results and Discussion

### 3.1 Single Factor Analysis of Phenol Adsorption by the CGFS-AFAC-800 Activated Material

#### 3.1.1 Comparison of Adsorption Effect under Different Activated Material Dosages

In order to explore the effect of activated material dosage on the adsorption effect, the activated material samples with a dosage of 0.1, 0.25, 0.5, 0.75, and 1.00 g were fixed under other conditions. The reaction time was set at 60 min, the temperature at 30 °C, the pH at 3, and the H_2_O_2_ concentration at 10 mM.

The adsorption effect of dosage on phenol is shown in Figure 2. As the dosage increases from 0.1 to 0.5 g, the adsorption capacity increases rapidly from 10.74 mg/g to 19.69 mg/g. This is mainly because with the increase of dosage, more adsorption sites are provided, and the synergistic effect with H_2_O_2_ is enhanced, so that the unit mass activated material captures more phenol molecules, and the total adsorption surface will expand accordingly, so the removal ratio of phenol in the solution continues to increase until the dosage is large enough. As the dosage is large enough, the phenol in the solution is almost completely removed and the removal rate is close to the limit. Therefore, the optimum dosage of the CGFS-AFAC-800 activated material for phenol adsorption was 0.75 g under the experimental conditions.

**Figure 2:**
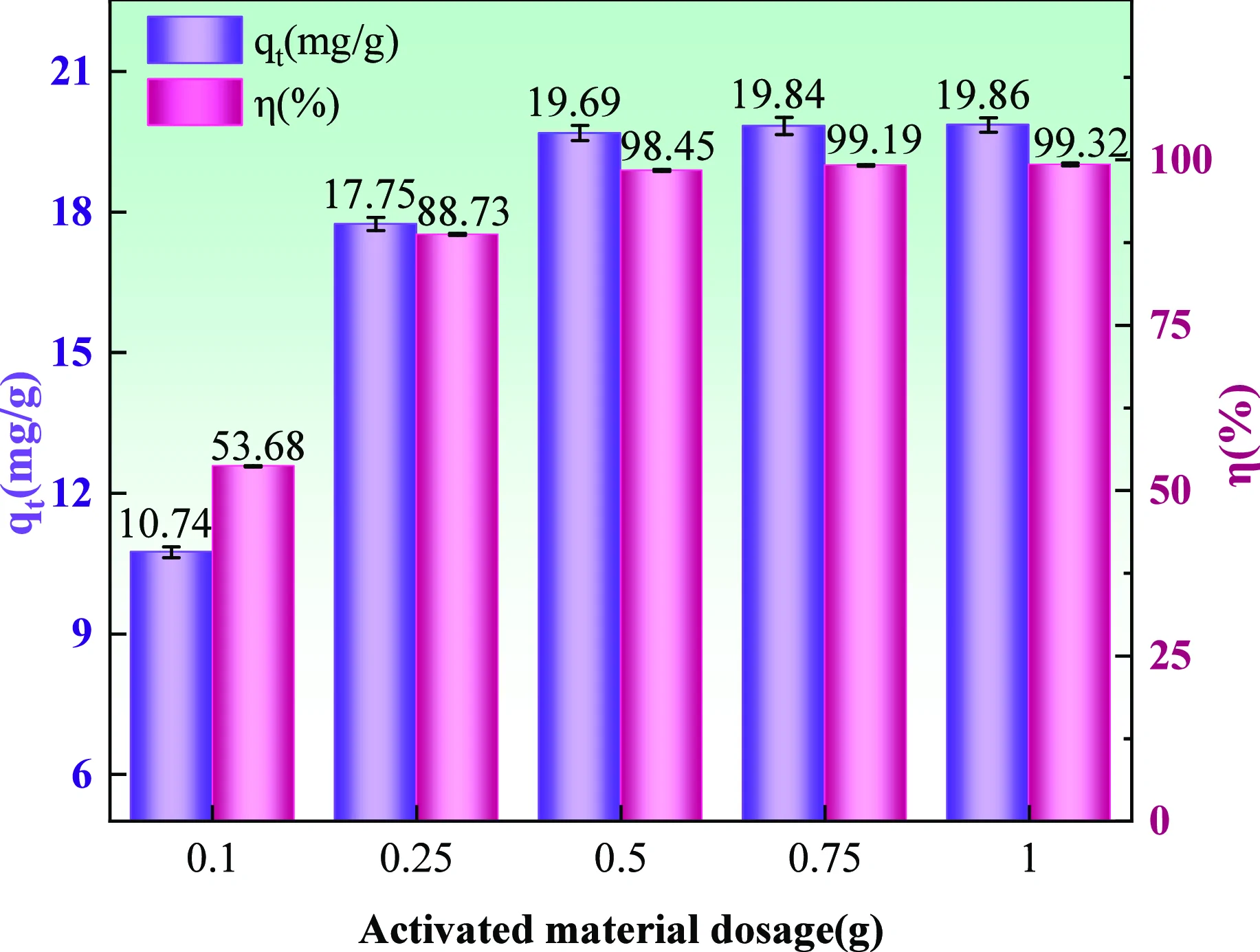
Effect of the activated material dosage on the adsorption effect.

#### 3.1.2 Comparison of the Adsorption Effect under Different Adsorption Times

In order to explore the effect of activated material adsorption time on the adsorption effect, 30, 60, 90, 120, 150, and 180 min were taken for adsorption in the experiment. Other conditions were fixed, the dosage was 0.75 g, the temperature was 30 °C, the pH was 3, and the H_2_O_2_ was 10 mM.

The effect of the adsorption time on the adsorption effect is shown in Figure 3. From 30 to 120 min, the adsorption capacity gradually increased from 12.99 mg/g to 13.26 mg/g, and the removal rate increased to the highest value of 99.36% at 120 min. This is mainly because at the initial stage of adsorption, there are a large number of available adsorption sites on the surface of the activated material, and phenol molecules can quickly diffuse to the surface of the activated material and be captured; at the same time, the adsorption–oxidation synergistic effect of H_2_O_2_ and adsorption gradually increased with time, which jointly promoted the increase of the adsorption capacity and removal rate. Therefore, under the experimental conditions, the optimum time for the adsorption of phenol by the CGFS-AFAC-800 activated material was 60 min.

**Figure 3:**
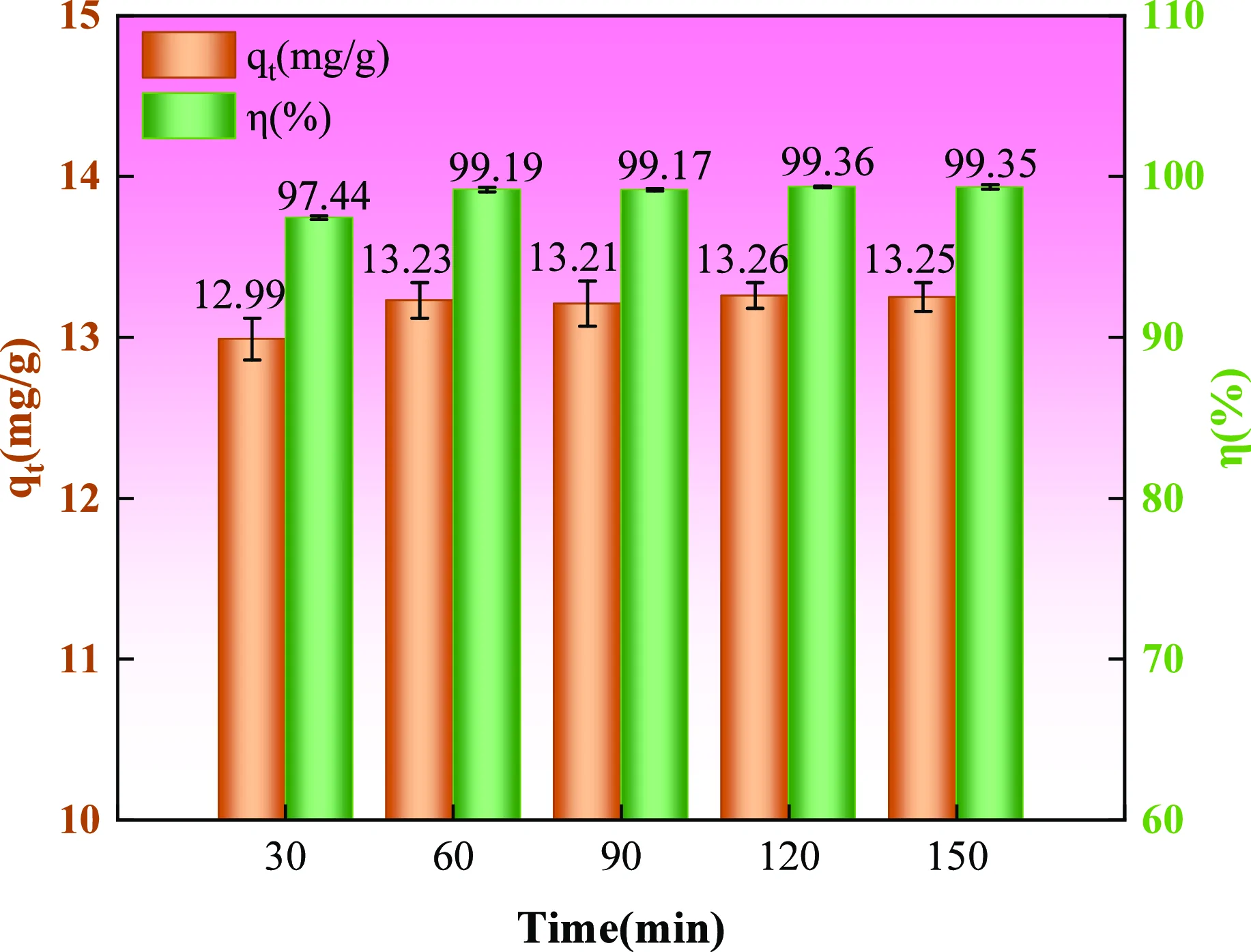
Effect of adsorption time on the adsorption effect.

#### 3.1.3 Comparison of the Adsorption Effect under Different Temperatures

In order to explore the effect of activated material adsorption temperature on the adsorption effect, the adsorption was carried out at 30, 40, 55, 70, and 85 °C, and other conditions were fixed. The dosage was 0.75 g, the time was 60 min, the pH was 3, and the H_2_O_2_ was 10 mM.

The effect of temperature on the adsorption effect is shown in Figure 4. At 25–40 °C, the adsorption capacity was maintained at 13.23 mg/g, and the removal rate reached about 99.20%. This is mainly because at 25–40 °C, the molecular kinetic energy is moderate, which not only promotes the diffusion and adsorption of phenol molecules to the surface of the activated material but also maintains the stability of H_2_O_2_, making it synergistic with the activated material and enhancing the activity of the adsorption site, thereby maximizing the removal effect. Therefore, under the experimental conditions, the optimum temperature for the adsorption of phenol by CGFS-AFAC-800 activated material was 30 °C.

**Figure 4:**
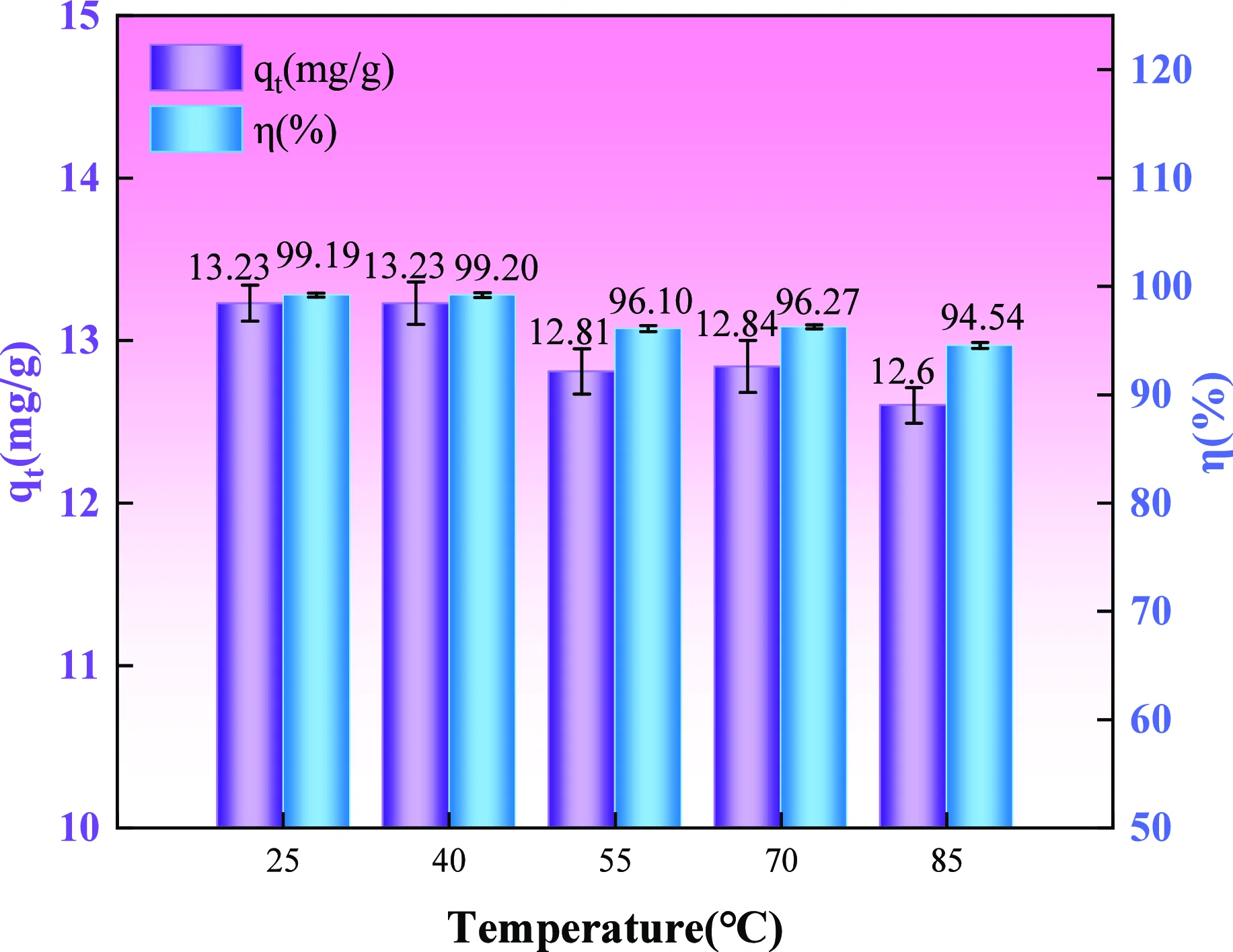
Effect of temperature on the adsorption effect.

#### 3.1.4 Comparison of the Adsorption Effect under Different Initial pH

In order to explore the effect of the initial pH of the solution on the adsorption effect, the pH of the phenol solution was adjusted with a concentration of 0.1 mol/L HCl solution and NaOH solution in the experiment, so that the pH of the phenol solution was 3, 5, 7, 9, and 11, and other conditions were fixed. The dosage was 0.75 g, the time was 60 min, the temperature was 30 °C, and the H_2_O_2_ was 10 mM.

The effect of initial pH of the solution on the adsorption effect is shown in Figure 5. As pH = 3, the adsorption effect is the best, the adsorption capacity is 13.31 mg/g, and the removal rate is 99.82%. This is mainly because when the solution is acidic, phenol mainly exists in the molecular state, and the molecular state phenol is captured by the activated material through π–π stacking. As the solution is alkaline, a large amount of phenol is dissociated into phenol anion C_6_H_5_O^–^. If the surface of the activated material is negatively charged due to the increase of pH, it will produce electrostatic repulsion with the phenol anion and hinder the adsorption. Therefore, under the experimental conditions, the optimum initial pH of the solution for the adsorption of phenol by the CGFS-AFAC-800 activated material was 3.

**Figure 5:**
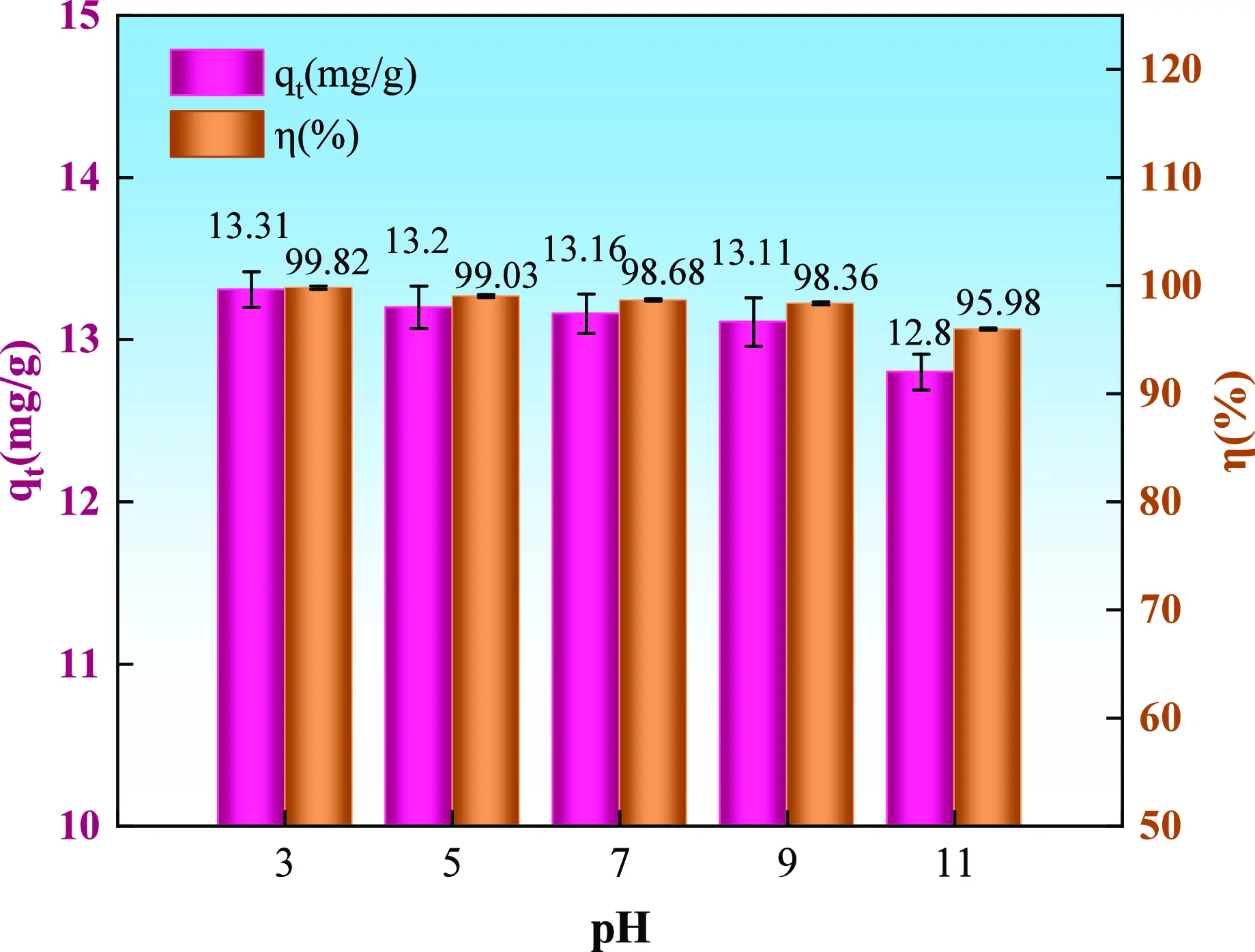
Effect of the initial pH of solution on the adsorption effect.

#### 3.1.5 Comparison of the Adsorption Effect under Different H_2_O_2_ Dosages

In order to explore the effect of the H_2_O_2_ dosage on the adsorption effect, the dosage of H_2_O_2_ in the experiment was 0, 5, 10, 15, and 20 mM, respectively, and other conditions were fixed. The dosage was 0.75 g, the reaction time was set at 60 min, the temperature at 30 °C, and the pH at 3.

The effect of the H_2_O_2_ dosage on the adsorption effect is shown in Figure 6. With the increase of H_2_O_2_ concentration from 0 mM to 20 mM, the unit adsorption capacity *q*_*t*_ of CGFS-AFAC-800 for phenol was stable between 13.15 and 13.23 mg/g, and the phenol removal rate was maintained in the range of 98.62–99.20%. The values of *q*_*t*_ and the removal rate corresponding to the concentration of each group fluctuated very little, and the data difference between different gradients fell within the standard deviation range of parallel experiments, with no statistically significant improvement. Only when the dosage of H_2_O_2_ was 10 mM did the treatment effect of the system show a small peak, and *q*_*t*_ and the removal rate reached the maximum value in this experiment. As the concentration of H_2_O_2_ was further increased to 15 mM and 20 mM, the adsorption capacity and removal efficiency decreased slightly, showing no continuous synergistic trend. This is mainly due to the fact that when the dosage of H_2_O_2_ was 0 mM, the physical/chemical adsorption only depends on the pore retention and surface functional groups of the activated material, and the adsorption sites were limited. As the amount of H_2_O_2_ was 10 mM, it had a synergistic effect on the activated material. Therefore, under the experimental conditions, the optimal H_2_O_2_ dosage for the adsorption of phenol by the CGFS-AFAC-800 activated material was 10 mM.

**Figure 6:**
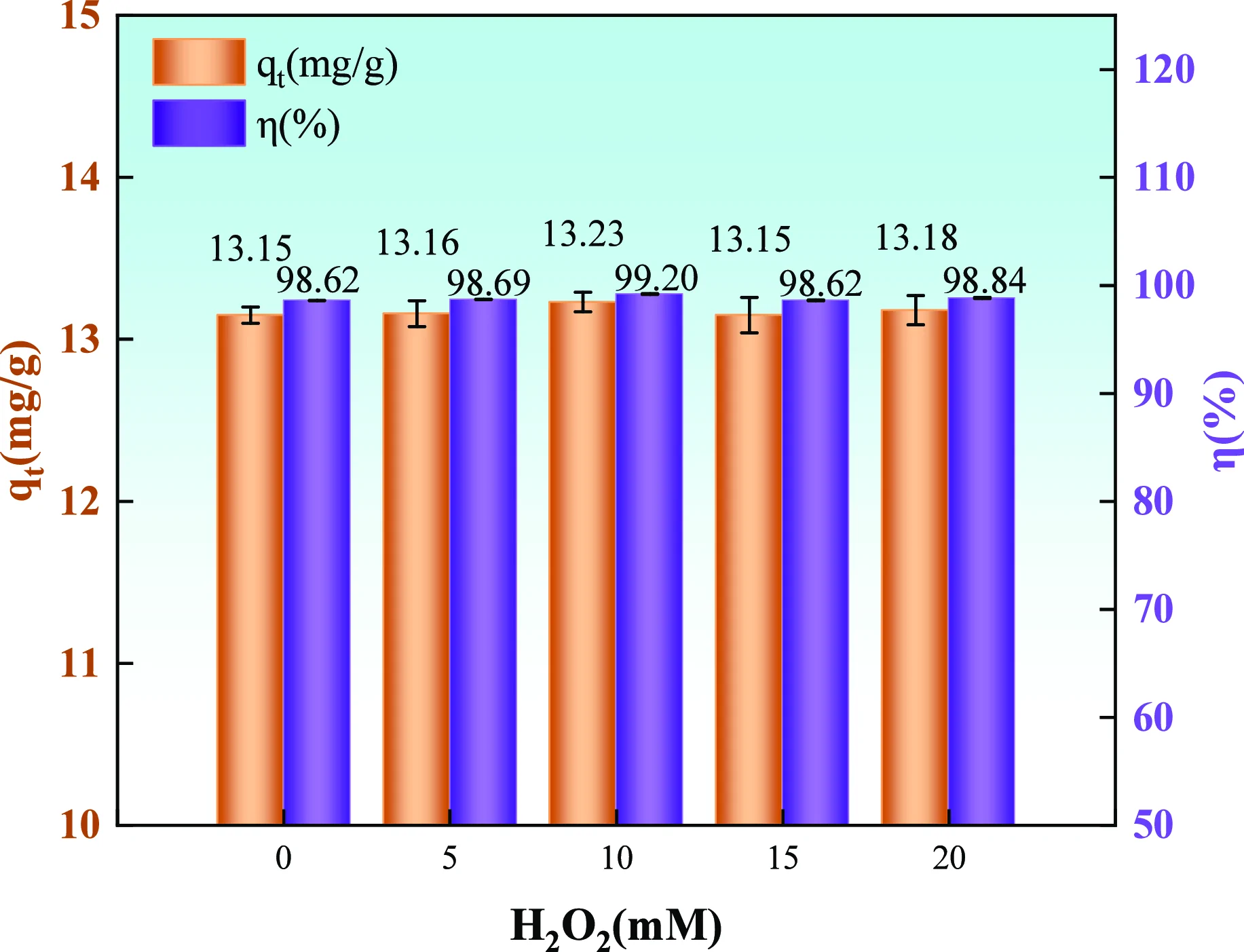
Effect of H_2_O_2_ dosage on the adsorption effect.

Through the above experimental data, the optimum conditions for the adsorption of phenol by the CGFS-AFAC-800 activated material when treating 100 mL of phenol solution with a concentration of 100 mg/L were obtained as follows: CGFS-AFAC-800 activated material dosage 0.75 g, time 60 min, temperature 30 °C, initial solution pH = 3, H_2_O_2_ dosage 10 mM.

### 3.2 Analysis of Orthogonal Test Results of Phenol Adsorption by the CGFS-AFAC-800 Activated Material

On the basis of the parameter interval determined by a single factor, the removal rate of phenol was taken as the main assessment target, and the orthogonal test of five factors and four levels was carried out. The orthogonal test scheme and experimental results are shown in Table 2.

**Table 2:** Orthogonal Scheme and Experimental Results

	level factor					
order number	A (g)	B (min)	C (°C)	D	E (mM)	removal rate (%)
1	0.25	30	30	1	0	91.95
2	0.25	60	40	3	5	95.01
3	0.25	90	55	5	10	88.94
4	0.25	120	70	7	15	82.36
5	0.5	30	40	5	15	95.86
6	0.5	60	30	7	10	97.71
7	0.5	90	70	1	5	98.6
8	0.5	120	55	3	0	99.46
9	0.75	30	55	7	5	96.21
10	0.75	60	70	5	0	97.45
11	0.75	90	30	3	15	99.58
12	0.75	120	40	1	10	99.90
13	1.0	30	70	3	10	99.26
14	1.0	60	55	1	15	99.60
15	1.0	90	40	7	0	99.66
16	1.0	120	30	5	5	99.85

As shown in Table 2, the removal rate of phenol by activated materials was up to 99.90%. The range analysis was performed on the table described above, and the results are shown in Table 3.

**Table 3:** Range Analysis of the Orthogonal Test^a^

	removal rate (%)					
factor	*E*_1_	*E*_2_	*E*_3_	*E*_4_	*R*	best
A	89.57	97.91	98.29	99.59	10.03	A_4_
B	95.82	97.44	96.70	95.39	2.05	B_2_
C	97.27	97.61	96.05	94.42	3.19	C_2_
D	97.51	98.33	95.53	93.99	4.34	D_2_
E	97.13	97.42	96.45	94.35	3.07	E_2_

a *E*_1_, *E*_2_, *E*_3_, and *E*_4_ represent four levels, and A, B, C, D, and E represent five factors.

The range analysis results of the removal rate of phenol by the CGFS-AFAC-800 activated material are shown in Table 3 and Figure 7. The order of the influence of five factors on the removal rate of phenol was activated material dosage > initial pH > reaction temperature > H_2_O_2_ dosage > adsorption time. Range analysis results showed that the dosage of the activated material imposed the strongest impact on the removal rate, with an *R* value of 10.03; the fourth level of this factor achieved an optimal removal effect. By contrast, the adsorption time had the smallest range *R* of 2.05, with its second level being the most appropriate condition. The *R* value of the adsorption temperature was 3.19, and the second level was selected as the optimal level. The solution pH recorded a range *R* of 4.34, and the second level yielded the best removal performance. As for the H_2_O_2_ dosage, its range *R* reached 3.07, with the second level confirmed as the best parameter.

**Figure 7:**
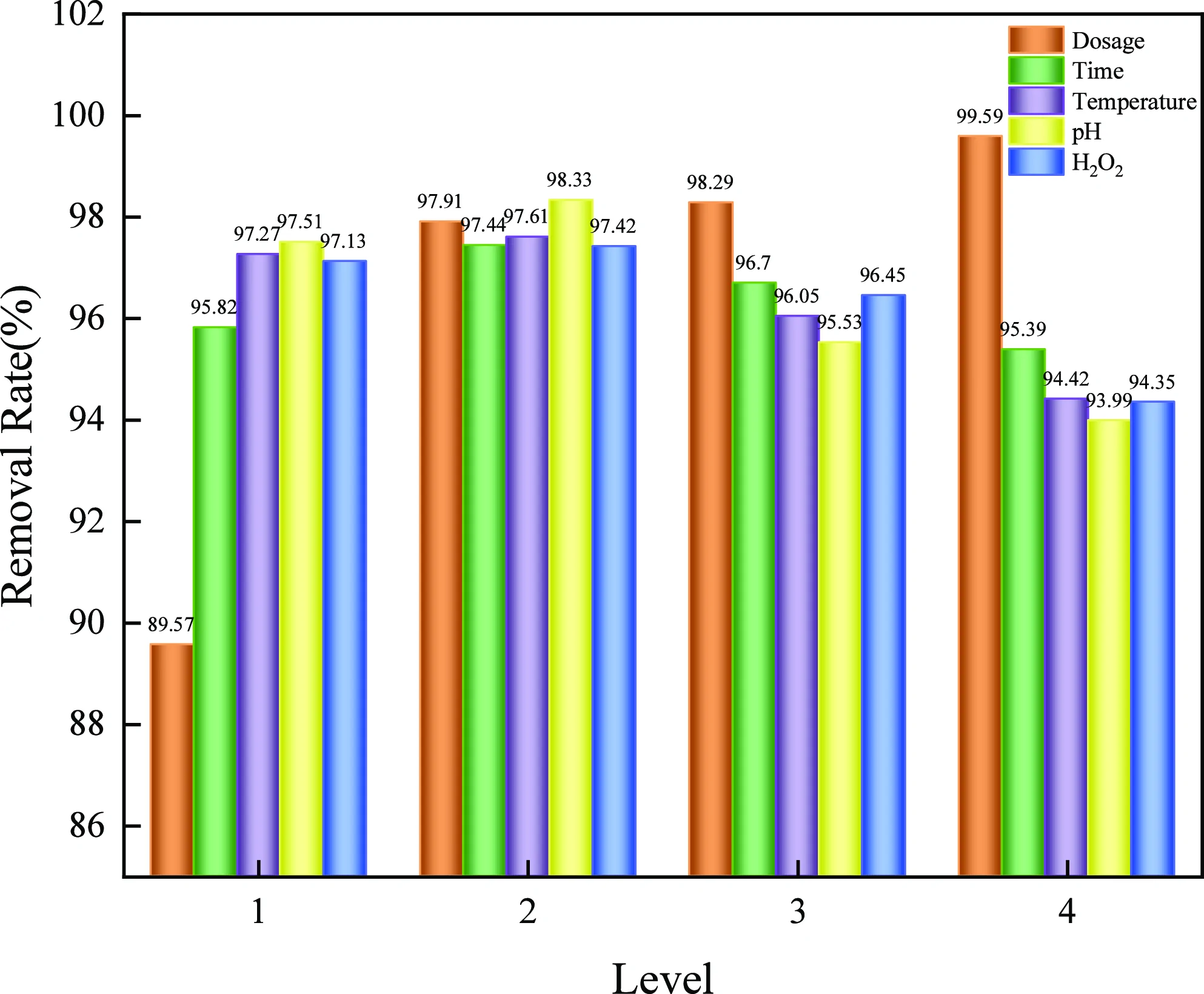
Range analysis of the orthogonal test.

The single factor experiment only examined the independent influence of a single variable, while the orthogonal experiment also covered the coupling interaction between multiple factors. Comprehensive analysis showed that the best solution was *A*_4_–*B*_2_–*C*_2_–*D*_2_–*E*_2_. The specific parameters of the optimal reaction system were set as follows: the reaction temperature was controlled at 40 °C, the initial pH of the solution was adjusted to 3, the dosage of the CGFS-AFAC-800 activated material was 1.0 g, the concentration of H_2_O_2_ was 5 mM, and the reaction duration was 60 min.

### 3.3 Adsorption Mechanism

#### 3.3.1 Adsorption Kinetics Analysis of CGFS-AFAC-800

The change is shown in Figure 8(a). Two successive stages can be identified throughout the entire adsorption process. During the early reaction period, the amount of phenol adsorbed by CGFS-AFAC-800 increased sharply, with the adsorption capacity reaching a peak of 13.22 mg/g at 60 min. With an extended reaction time, the rising rate of phenol adsorption capacity showed varying degrees of slowdown, and the adsorption reaction eventually reached equilibrium at 90 min. Finally, the maximum saturated adsorption capacity of CGFS-AFAC-800 for phenol solution with a concentration of 100 mg/L was 13.22 mg/g. The adsorption process shows such a trend because in the later stage of the reaction, the available adsorption sites on the activated material are less and less, and the phenol concentration decreases and the concentration gradient becomes smaller; therefore, the adsorption reaction is carried out in a dynamic equilibrium state.

**Figure 8:**
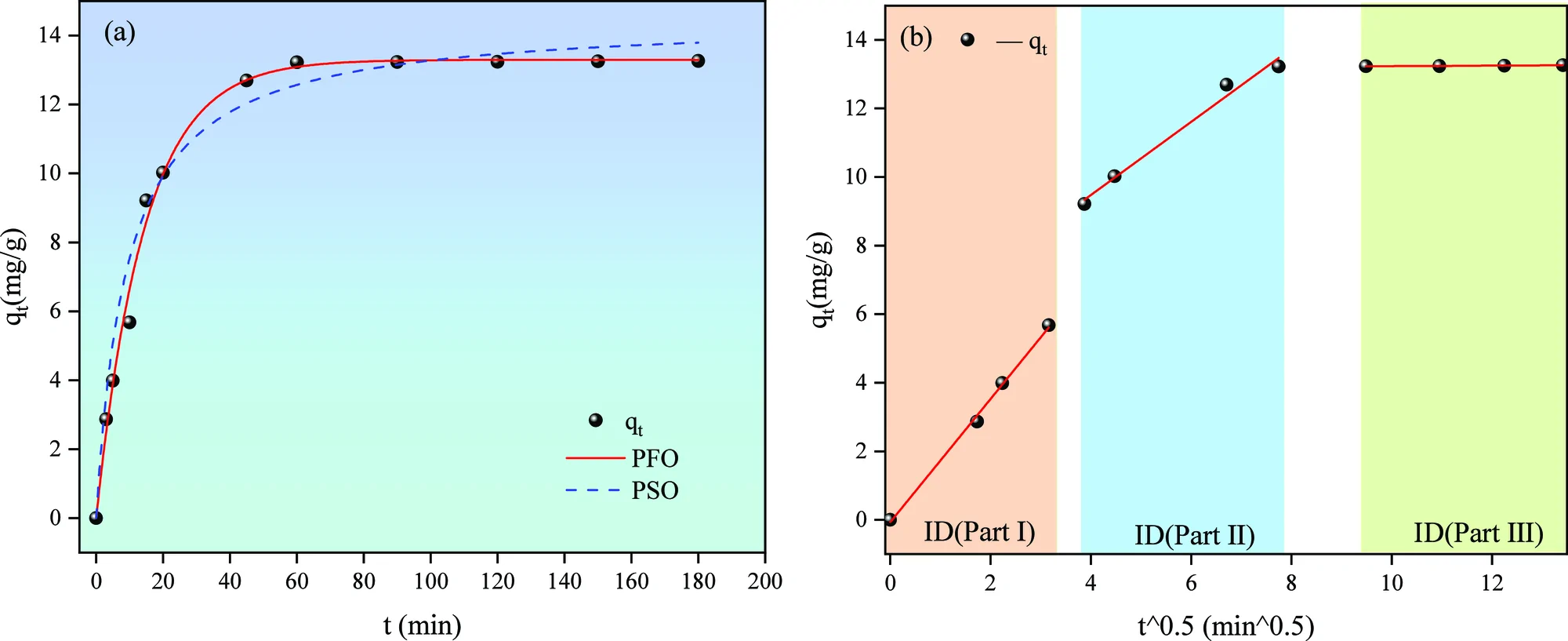
Adsorption kinetics of phenol on CGFS-AFAC-800: (a) The change of adsorption capacity with time and the fitting results of the PFO model and PSO model. (b) ID model fitting results.

The pseudo-first-order kinetic (PFO) model and the pseudo-second-order kinetic (PSO) model were used to describe the adsorption process of phenol by CGFS-AFAC-800. The model fitting is shown in Figure 8(a) and (b), respectively.

The relevant kinetic parameters are listed in Table 4. Specifically, the parameter *q*_e,cal_ (in mg/g) represents the theoretical equilibrium adsorption capacity yielded by the nonlinear fitting calculation. Constants *K*_1_ and *K*_2_ refer to the adsorption rate coefficients matching the first-order and second-order kinetic models, respectively. According to Table 4, the correlation coefficient *R* ^2^ (0.9937) fitted by the PFO model is higher than that of the PSO model (0.9661) on the whole, and the theoretical equilibrium adsorption capacity *q*_e,cal_ calculated via the PFO model exhibits better consistency with the experimentally obtained *q*_e,exp_ value. As a result, the PFO model is more capable of describing the phenol adsorption behavior of the CGFS-AFAC-800. On the basis of the hypothesis of the PFO kinetic model, the entire adsorption process is mainly dominated by the diffusion step. In the PSO theory, the adsorption rate is mainly controlled by chemical adsorption.^25^ Therefore, the adsorption type of phenol on CGFS-AFAC-800 is mainly physical diffusion or surface diffusion, mainly because the activated material prepared by the alkali fusion method destroys part of the carbon structure and reduces the density of functional groups. Therefore, in this study, the alkali fusion method was used to treat the coal gasification fine slag, which may lead to the reduction of surface chemically active sites, and the adsorption mechanism was dominated by physical action. Therefore, the pseudo-first-order kinetic model (PFO) had a better fitting effect, which was in line with the adsorption process dominated by diffusion or surface coverage.

**Table 4:** Adsorption Kinetic Parameters of Phenol on CGFS-AFAC-800

		parameters		
kinetics model		*q*_e_ (mg·g^–1^)	*K*_i_ (min^–1^)	*R* ^2^
pseudo-first-order		13.30	0.07	0.9937
pseudo-second-order		14.50	0.01	0.9661

		*C* (mg·g^–1^)	*K*_id_ (mg·g^–1^·min^–1^)	*R* ^2^
intraparticle-diffusion model	part I	0	1.80	0.9964
	part II	5.21	1.07	0.9757
	part III	13.16	0.01	0.9296

Figure 8(b) shows the fitting results of the intraparticle–diffusion model for the adsorption process of phenol by CGFS-AFAC-800. As shown in Figure 8 and Table 4, *K*_id_ is the diffusion rate constant of the ID model and C is a constant related to the thickness of the boundary layer. The adsorption process can be divided into three stages: the first stage is the membrane diffusion stage, corresponding to 0–10 min, the phenol molecules rapidly diffuse to the outer surface of CGFS-AFAC-800, showing a higher diffusion rate constant (*K*_d1_); the second stage is the intraparticle diffusion stage, corresponding to 10–60 min, the molecules gradually enter the pores, and the diffusion rate slows down (*K*_d2_). The third stage is the equilibrium stage, corresponding to 60–180 min, the adsorption sites tend to be saturated, and the diffusion rate is further reduced (*K*_d3_). Therefore, although the pseudo-first-order kinetic model (PFO) fits the experimental data well, it is only an apparent model and cannot fully reflect the adsorption mechanism, which must be further analyzed in combination with the diffusion model.

#### 3.3.2 Isothermal Adsorption Experiment of CGFS-AFAC-800

A series of isothermal adsorption tests were carried out to explore the variation relationship between the equilibrium adsorption capacity *q*_e_ of CGFS-AFAC-800 and the equilibrium solution concentration *C*_e_. The initial mass concentration of phenol solution was adjusted to range from 50 to 150 mg/L, with a fixed solution volume of 100 mL for each adsorption reaction. The CGFS-AFAC-800 sample was used as the activated material, the dosage was 1.0 g, the reaction time was 60 min, and the reaction temperature was set to 25, 30, and 35 °C, respectively. The equilibrium concentration and equilibrium adsorption capacity are shown in Figure 9. With the increase of *C*_e_, the adsorption capacity *q*_e_ of CGFS-AFAC-800 for phenol showed an overall upward trend, especially at a low equilibrium concentration of the solution, and the increase was slowed down when the equilibrium concentration increased. As the concentration was low, the higher the temperature is, the greater the adsorption capacity was at 25, 30, and 35 °C. This observation further verifies that the adsorption process proceeded via an endothermic reaction mechanism, and increasing temperature exerted a positive promoting effect on phenol removal.

**Figure 9:**
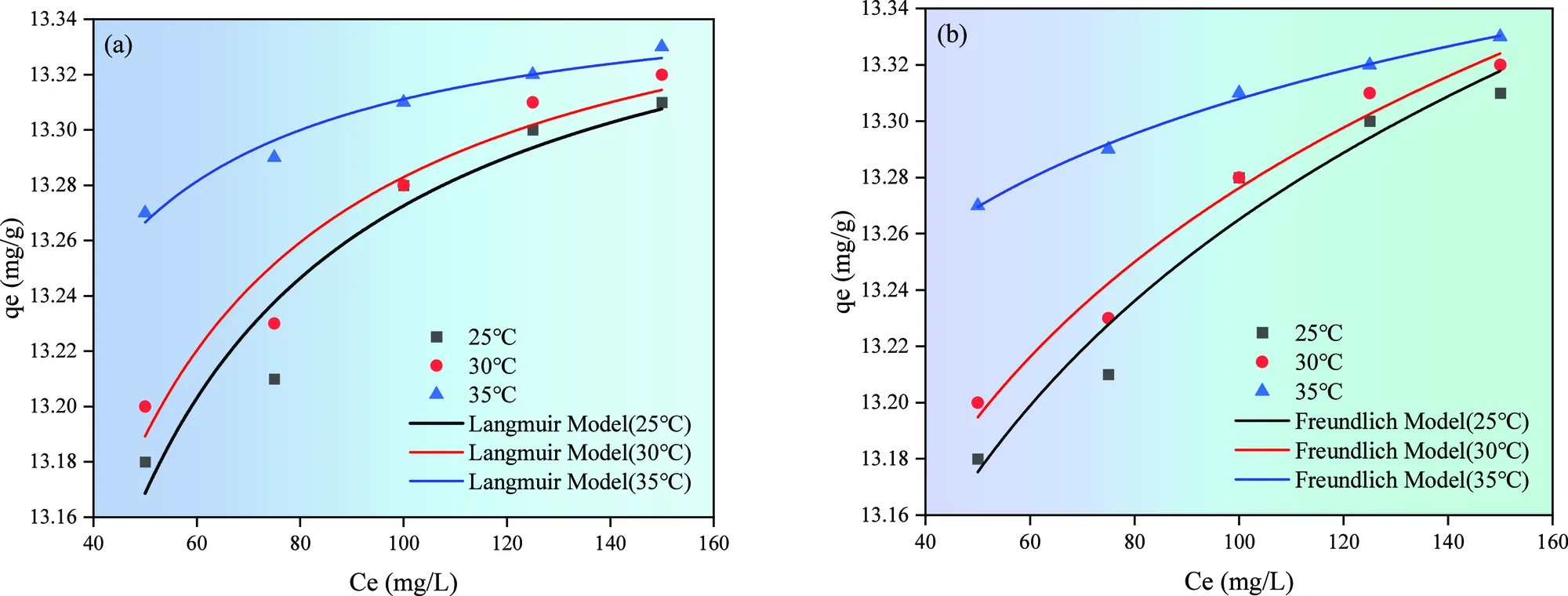
Adsorption isotherms of CGFS-AFAC-800 for phenol wastewater: (a) Langmuir model fitting results and (b) Freundlich model fitting results.

To more accurately characterize the phenol adsorption process onto CGFS-AFAC-800, Langmuir and Freundlich isotherm models were selected for the fitting analysis. The Langmuir model was used to discuss the adsorption equilibrium in the process of monolayer adsorption on a homogeneous surface. The basic characteristics were expressed by separation factor *R*_L_. The Freundlich model is used to describe the multilayer adsorption process on the surface. The distribution of the two isotherms obtained by fitting is shown in Figure 9, and the fitting parameters are listed in Table 5.

**Table 5:** Isothermal Adsorption Parameters of Phenol on CGFS-AFAC-800

		CGFS-AFAC-800		
isotherms	parameters	25 °C	30 °C	35 °C
Langmuir	*q*_m_ (mg/g)	13.3783	13.378	13.356
	*K*_L_ (L/mg)	1.25598	1.39727	2.96948
	*R*_L_	0.0005–0.0157	0.0005–0.0141	0.0002–0.0007
	*R* ^2^	0.8998	0.9143	0.9611
Freundlich	*K*_F_ (mg/g(L/mg)^1/*n*^)	12.68	12.74	13.05
	*n*	102.15	112.74	239.81
	*R* ^2^	0.9343	0.9667	0.9950

Figure 9(a) and (b) shows the nonlinear fitting results of Langmuir and Freundlich isothermal adsorption models, respectively. Table 5 is the isothermal adsorption parameters of the two models. The adsorption capacity increases with the increase of concentration, this is because an elevated concentration disparity can deliver a stronger driving force, allowing the particles to overcome the mass transfer resistance, so that more phenol enters the activated material CGFS-AFAC-800 and binds to the adsorption site.

Comparing the fitting of the two models, the experimental results are more consistent with the Freundlich model. At 25, 30, and 35 °C, the fitting correlation coefficients *R* ^2^ of the Freundlich model are 0.9343, 0.9667, and 0.9950, respectively, while the *R* ^2^ of the Langmuir model is only about 0.9. The surface of the alkali fusion coal gasification fine slag is complex and the energy distribution is uneven. Phenol has multilayer adsorption on it, and the Freundlich model can more truly reflect this process; so, *R* ^2^ is higher. This indicates that the Freundlich model is more suitable for describing the adsorption process of phenol by CGFS-AFAC-800, and the value of the nonuniformity factor *n* exceeds 1, which also reflects the good adsorption process.^28^ After alkali fusion activation, nepheline and other phases are formed on the surface of coal gasification fine slag, and the surface chemical properties are greatly different. These different phases provide adsorption sites of different energy levels, which is in line with the core hypothesis of the Freundlich model. The separation factor of the Langmuir model is 0 < *R*_L_ < 1, and the nonuniformity factor of the Freundlich model is *n* >1, indicating an ideal and efficient adsorption process during adsorption.^29,30^ In addition, the theoretical adsorption capacities obtained by the Freundlich model at three temperatures from low to high were 12.68, 12.74, and 13.05 mg/g, respectively, which were close to the experimental values (13.26, 13.27, and 13.30 mg/g), again proving the reliability of the Freundlich model fitting results.

#### 3.3.3 Adsorption Thermodynamic Analysis of CGFS-AFAC-800

The calculation of thermodynamic parameters needs to be combined with the experimental data of isothermal adsorption. The calculation methods of three important parameters, standard Gibbs free energy (Δ*G*°), standard adsorption enthalpy (Δ*H*°), and standard adsorption entropy (Δ*S*°), are shown in Formulas [Disp-formula eq10], 11, and 12. The obtained adsorption thermodynamic parameters are summarized in Table 6.

**Table 6:** Thermodynamic Parameters of Adsorption of Phenol by CGFS-AFAC-800

T (°C)	ln *K*°	Δ*G*° (kJ·mol^–1^)	Δ*H*° (kJ·mol^–1^)	Δ*S*° (kJ·mol^–1^·K)
25	3.43	–8.49		
30	3.41	–8.58	6.54	6.12
35	3.34	–8.56		

At 25, 30, and 35 °C, Δ*G*° was negative (−8.49, −8.58, and −8.56 kJ·mol^–1^), indicating that the adsorption reaction of phenol by CGFS-AFAC-800 was a spontaneous process. A gradual decline in ln *K*° can be observed with increasing temperature, accompanied by a growing absolute value of Δ*G*°. This variation trend confirms that the adsorption behavior features higher spontaneity at elevated temperatures,^31^ and the physical adsorption free-energy change is generally −20–0 kJ·mol^–1^. Therefore, it can also be judged that the adsorption process of phenol by CGFS-AFAC-800 is physical adsorption. Δ*H*° > 0 indicates that the reaction process is an endothermic reaction, and its absolute value is less than 40 kJ·mol^–1^, indicating that physical adsorption plays a significant role in the adsorption of phenol by CGFS-AFAC-800. Δ*S*° > 0 corresponds to the entropy increase process, and the degree of confusion at the solid–liquid interface increases.^32^

## 4 Conclusions

In this study, a CGFS-AFAC-800 activated material was prepared by the NaOH alkali fusion method, and the efficient removal and mechanism of phenol wastewater were studied. The following conclusions were obtained:

(1) The orthogonal test was further optimized to obtain the optimal scheme for the dosage of 10 g/L, time 60 min, temperature 40 °C, pH = 3, H_2_O_2_ dosage 5 mM, and phenol removal rate up to 99.90%.
(2) The adsorption mechanism study showed that the adsorption process of phenol by CGFS-AFAC-800 conformed to the pseudo-first-order (PFO) kinetic model (*R* ^2^ = 0.9937). The adsorption rate was controlled by membrane diffusion and intraparticle diffusion, and adsorption equilibrium could be reached within 60 min. The maximum saturated adsorption capacity was 13.22 mg/g.
(3) The isothermal adsorption behavior was more consistent with the Freundlich model (*R* ^2^ = 0.9950 at 35 °C), which confirmed that the multilayer adsorption of phenol on the heterogeneous surface of the material surface occurred. The nonuniformity factor was *n* >1, and the adsorption process was efficient and feasible.
(4) The thermodynamic parameters show that Δ*G*° is −8.49 to −8.58 kJ·mol^–1^, Δ*H*° = 6.54 kJ·mol^–1^, and Δ*S*° = 6.12 kJ·mol^–1^·K^–1^, indicating that the adsorption reaction is a spontaneous and endothermic physical adsorption process and is driven by the entropy increase effect.

## Data Availability

The data that support the findings of this study are available from the corresponding author upon reasonable request.
